# Antidepressants in bipolar depression: an enduring controversy

**DOI:** 10.1186/s40345-018-0133-9

**Published:** 2018-12-01

**Authors:** Michael J. Gitlin

**Affiliations:** Department of Psychiatry, Geffen School of Medicine at UCLA, Los Angeles, CA USA

**Keywords:** Bipolar depression, Antidepressants, Treatment emergent affective switch

## Abstract

The proper place and the optimal use of antidepressants in treating bipolar depression continues to be an area of great interest and greater controversy with passionate opinions more common than good studies. Even the handful of meta-analyses in the area disagree with each other. Overall, the evidence that antidepressants are effective in treating bipolar depression is weak. Additionally, many experts and clinicians worry greatly about the capacity of antidepressants to cause affective switching or mood destabilization. Yet, in short term controlled studies, with most patients also taking mood stabilizers, antidepressants are not associated with switches into mania/hypomania. Evidence of cycle acceleration with antidepressants primarily reflects treatment with older antidepressants, e.g., tricyclics. Similar evidence with modern antidepressants such as selective serotonin reuptake inhibitors (SSRIs) is lacking. The key questions should not be: are antidepressants effective in bipolar depression?; And: do antidepressants worsen the course of bipolar disorder? Rather, the question should be focused on subgroups: for which patients are antidepressants helpful and safe, and for which patients will they be harmful? Predictors of affective switching with antidepressants include: bipolar I disorder (vs. bipolar II), mixed features during depression, tricyclics vs. modern antidepressants, rapid cycling and possibly a history of drug abuse, especially stimulant abuse. Additionally, a number of recent studies have demonstrated both the safety and efficacy of antidepressant monotherapy in treating bipolar II depression. Finally, a subgroup of bipolar individuals need antidepressants in addition to mood stabilizers as part of an optimal maintenance treatment regimen.

## Background

Among the three phases of treatment of bipolar disorder-acute mania, maintenance treatment and acute depression, the latter poses the greatest difficulty in a variety of ways. Assuredly, this is due to a number of factors including: the relative paucity of studies in the area (although more data have emerged in the last number of years); the discrepancy in conclusions between meta-analyses; and the heated debates on the specific role of antidepressants in treating bipolar depression. On the latter issue—the proper place of antidepressants for bipolar depression—experts view the same handful of controlled studies and derive markedly diverse clinical recommendations with many if not most papers recommending caution (or sometimes extreme caution) in their use. A thoughtful Consensus statement from the International Society for Bipolar Disorders (ISBD) on the topic published 5 years ago concluded that “the available evidence, both the value and the risks of antidepressant treatment in bipolar disorder is remarkably limited, and much of it is methodologically weak” (Pacchiarotti et al. 2013). This paper will briefly review the overall topic of bipolar depression, summarize the other available treatments and then provide an update on antidepressants in bipolar depression.

## Bipolar depression

Even though polarity—the presence or absence of manic/hypomanic episodes—is the defining feature in our classification of primary mood disorders, distinguishing unipolar from bipolar disorder, depression is the dominant pole. Overall, including both bipolar I and II patients, bipolar individuals spend three times as many weeks depressed as they do manic/hypomanic (Baldessarini et al. 2010). In bipolar II patients, depression may be even more dominant. As an example, one study found a 37:1 ratio of depressive to hypomanic weeks in bipolar II patients over a 13 year naturalistic follow-up period (Judd et al. 2003).

The range of symptoms seen in unipolar and bipolar depression do not differ, consistent with our classification systems that use the same diagnostic criteria for both syndromes. Some symptoms are more common in bipolar depression, including hypersomnia, hyperphagia/weight gain, psychomotor retardation and psychotic symptoms (Goodwin and Jamison 2007; Caspar et al. 1985). However, no single symptom or group of symptoms reliably distinguishes unipolar from bipolar depression.

## First line/classic treatments for acute bipolar depression

The consensus in the field is that non-antidepressant treatments should be considered as monotherapy before using antidepressants to treat bipolar depression (Pacchiarotti et al. 2013). (In contrast, however, multiple studies have shown that clinicians prescribe antidepressants with and without mood stabilizers far more frequently than Expert Consensuses and Practice Guidelines would suggest—Baldessarini et al. 2007; Viktorin et al. 2014). The medications with the best data from randomized controlled trials are second generation antipsychotics (SGAs). Although this partly reflects the capacity of pharmaceutical firms to fund large scale studies, the consistent efficacy across multiple studies suggests true antidepressant activity. The two SGAs with the best data are quetiapine and lurasidone, both of which demonstrated efficacy in at least two large scale placebo controlled studies (Sanford and Keating 2012; Loebel et al. 2014a, b; Rajagopolan et al. 2016; Pikolov et al. 2017). Olanzapine (Tohen et al. 2003) and cariprazine (Durgan et al. 2016) have also shown efficacy in at least one double-blind study each. Not all SGAs have shown consistent efficacy. Ziprasidone was ineffective in one controlled study (Sachs et al. 2011) while aripiprazole failed its registrational trials (Thase et al. 2008) (although excessively high doses may partly explain the latter results). In general, optimal doses of SGAs for bipolar depression are substantially lower than the doses used to treat acute psychosis or acute mania (Bartoli et al. 2017).

## Antidepressants in bipolar depression: conceptual concerns

When antidepressants are prescribed for unipolar depression and mood stabilizers for bipolar depression, the only concerns are those of efficacy (or lack thereof) and side effects. There are no credible reasons to worry about the proposed treatment exacerbating the disorder. (I am ignoring the potential issue of worsening suicidality in adolescents and young adults treated with antidepressants since it is both controversial and infrequently seen). The two added concerns when treating bipolar depression with antidepressants are switching into mania/hypomania and/or the induction of rapid cycling. Switching into mania/hypomania, now called treatment emergent affective switch (TEAS) (Tohen et al. 2009) has been well recognized since the first use of antidepressants to treat bipolar depression. Yet, without proper control groups, assigning causality to the use of an antidepressant and the emergence of mania/hypomania is fraught with difficulties since a substantial portion of bipolar patients show a naturalistic pattern of depression followed by mania/hypomania *without* treatment. In any individual case, it is impossible to know whether the post-depression mania/hypomania is due to the antidepressant prescribed or to the natural history of the disorder. In order to deal with this conundrum, a reasonable (although arbitrary) definition of definite TEAS would be the emergence of a syndromal mania/hypomania within 8 weeks of either the initiation of the antidepressant or an increase in its dose (Tohen et al. 2009). Likely or possible TEAS should be considered if hypomanic symptoms not meeting syndromal criteria emerge within 12 weeks of antidepressant initiation or dose increase.

The second concern about negative effects of antidepressants in bipolar disorder is the evidence that antidepressants may be associated with altering/exacerbating the course of the illness for months or years. Varied terms have been ascribed to this phenomenon including: induction of rapid cycling, roughening the course of the disorder, cycle acceleration, increased affective lability and so forth. The core phenomenon expressed by all these terms is an increase in mood shifts per unit time (Miklowitz and Gitlin 2015). This may manifest by a true cycle acceleration—i.e., more DSM defined mood episodes over (e.g.) a 1 year time frame, or more within day and/or day to day rapid mood shifts and fewer euthymic days/weeks (which would not necessarily be captured by formal episode counting).

## Antidepressants for acute bipolar depression: a summary of the efficacy data and meta-analyses

Over the last decade, a number of reviews and meta-analyses have examined the relative small research literature on the efficacy of antidepressants in bipolar depression. Studies in this area included studies with either only bipolar I or a combination of bipolar I and bipolar II patients. (The data on treating bipolar II depression specifically will be reviewed below). Both narrative reviews (Licht et al. 2008) and meta-analyses differ in their conclusions, reflecting subtle but important differences in study inclusion/exclusion criteria and statistical techniques. As examples, Sidor and MacQueen (2012) included 7 studies in their meta-analysis, McGirr et al. (2016) included 6 studies, while Vasquez et al. (2013) included 10 studies. Considering either response, remission or both, two of these meta-analyses found no significant efficacy of antidepressants in bipolar depression (Sidor and MacQueen 2012; McGirr et al. 2016) while another (Vasquez et al. 2013) found efficacy with a relative risk (RR) = 1.43 (CI 1.11–1.84, p < 0.006) and with a number to treat (NNT) = 6.2. Using a different approach, an additional analysis examined all studies comparing antidepressant efficacy in unipolar and bipolar depressed patients and found no difference in efficacy (pooled RR = 1.05, CI 0.96–1.15, p = 0.34) (Vasquez et al. 2011). Finally, a meta-analysis which compared antidepressants for bipolar depression to separate studies on antidepressants in unipolar depression found comparable efficacy (Vasquez et al. 2013).

Most, but not all bipolar subjects in the studies included within the meta-analyses were on mood stabilizers. Thus, the studies primarily examined the additive efficacy of antidepressants as opposed to antidepressant monotherapy. In the only double-blind, placebo-controlled study published in the last 30 years, paroxetine monotherapy was not more effective than placebo in treating bipolar I and II depression (McElroy et al. 2010). Critiques of this study includes a low (20 mg) fixed dose of paroxetine and very high placebo response and remission rates (53% and 55%). Of note, the most recent meta-analysis in this area found efficacy when antidepressants were added to second generation antipsychotics but not when added to classic mood stabilizers (lithium or anticonvulsants). When added to SGAs, antidepressants showed higher response (51% vs. 37%, p = 0.007; NNT = 7) and remission rates (45% vs. 31%) (McGirr et al. 2016). Whether this reflects additive efficacy from the SGAs or some other factor is unknown.

Thus, the only reasonable conclusion would be that, with the relative paucity of data available, the effectiveness of antidepressants, whether prescribed as monotherapy or adjunctive to mood stabilizers for bipolar depression is still unproven (Gitlin 2012).

## Antidepressants for acute bipolar depression: a summary of the switch data and meta-analyses

Most of the same reviews and meta-analyses also examined switch rates comparing antidepressants to placebo. All analyses agree that, in short-term studies, when antidepressants are added to mood stabilizers (in most of the patients), switch rates do not differ between antidepressants and placebo with p values = 0.89 and 0.93 (Sidor and MacQueen 2012; McGirr et al. 2016).

Of note, in the only monotherapy study using paroxetine as the antidepressant (McElroy et al. 2010) with bipolar I and bipolar II patients, switch rates in the short term (8 weeks) also did not differ between antidepressant and placebo (11% vs. 9%).

Although far from certain, it is likely that different antidepressant classes confer different vulnerability to switching (Peet 1994; Gijsman et al. 2004; Tondo et al. 2010; Vasquez et al. 2013). Tricyclics are generally considered to have the highest switch rate while selective serotonin reuptake inhibitors (SSRIs) and bupropion confer the lowest risk. Serotonin/norepinephrine reuptake inhibitors (SNRIs) (at least, venlafaxine) are associated with higher risks than SSRIs and bupropion (Vieta et al. 2002; Post et al. 2006). Whether this increased risk is shared with other SNRIs such as duloxetine and desmethylvenlafaxine is unknown. Switch rates with monoamine oxidase (MAO) inhibitors are generally considered to be less than with tricyclics although data supporting this are not available (Himmelhoch et al. 1991).

Surprisingly, definitive evidence from large studies and meta-analyses that mood stabilizers diminish the risk of TEAS is lacking (Sachs et al. 2007; Tondo et al. 2010) despite individual studies that seem to demonstrate the greater safety of antidepressants when added to mood stabilizers (Bottlender et al. 2001; Henry and Demotes-Mainard 2003). In a large, population based study (n = 3240), using a within-individual design in bipolar I patients, over a 3 month period, antidepressants were not associated with higher switch rates when added to mood stabilizers (hazard ratio [HR] = 0.79 [CI 0.54–1.15, ns]). In contrast, antidepressant monotherapy in the same individuals was associated with an almost three times increase in TEAS (HR = 2.83, [CI 1.12–7.19, p = 0.028]) (Viktorin et al. 2014).

## Cycle acceleration from antidepressants

As described above, aside from TEAS, cycle acceleration, first described decades ago, comprises the other major clinical concern when antidepressants are prescribed to bipolar patients. A number of studies from the tricyclic era seemed to suggest a link between antidepressant use and cycle acceleration (Wehr and Goodwin 1979; Kukopulos et al. 1980; Wehr et al. 1988; Altshuler et al. 1995). It is less clear whether this phenomenon is seen in any consistent way with more modern antidepressants-SSRIs, bupropion, SNRIs-especially if the patient is also on mood stabilizers (Bauer et al. 2006). In one study using modern antidepressants, an association was found between prescription of antidepressants and rapid cycling (Schneck et al. 2008). However, as noted earlier by Coryell et al. (2003), since episodes of depression (which then trigger the prescription of the antidepressant) often precede rapid cycling, the relationship between rapid cycling may be associative, not causal. Overall, only some of the prospective studies demonstrate a link between rapid cycling and antidepressant use (Carvalho et al. 2014).

## Treating bipolar II depression with antidepressants

The studies reviewed above examined either bipolar I depression or a mixture of bipolar I and II patients. As noted, bipolar II disorder may be far more dominated by depression compared to bipolar I patients (Judd et al. 2003). But, there are legitimate reasons to consider that the risk/benefit ratio of antidepressants in bipolar II patients may differ markedly from bipolar I patients. As one example, whereas when bipolar I patients switch, they do so almost equally into mania (45%) vs. hypomania (55%), bipolar II patients switch into hypomania 90% of the time (Bond et al. 2010). Additionally, whether all (mild) hypomanias need to be treated is debatable (Parker 2012). Finally, bipolar II patients demonstrate TEAS at approximately 50% the rate of bipolar I patients (Bond et al. 2010). Thus, switches with bipolar II patients are both less frequent and milder, diminishing the risk of antidepressant treatment considerably.

A corollary question is whether bipolar II depression can be safely and effectively treated with antidepressant monotherapy. A handful of recent studies have suggested both efficacy and safety of antidepressant monotherapy in short term studies in this population. (Amsterdam and Brunswick 2003; Amsterdam and Shults 2010; Amsterdam et al. 2010, 2015, 2016; Altshuler et al. 2017). In a recent study comparing venlafaxine to lithium, the SNRI showed greater efficacy with no differences in switch rates both in the acute study (12 weeks) and during a 6 month continuation study (Amsterdam et al. 2015, 2016). This is particularly noteworthy given that two prior studies (Vieta et al. 2002; Post et al. 2006) demonstrated higher switch rates with venlafaxine compared to an SSRI (in both studies) or bupropion (one study). In the largest double-blind, controlled study, no efficacy differences were seen in 142 bipolar II depressed patients randomized to sertraline, lithium, or lithium plus sertraline for 16 weeks (Altshuler et al. 2017). Additionally, no differences in switch rates were seen across the three groups. Of note, switch rates for the lithium group (19%) were identical to the rate in the sertraline treated group (20%), highlighting again the naturalistic rate of post-depression mania/hypomania. Also consistent with evidence from earlier studies, no switches to formal mania were seen among this bipolar II population. In subgroup analyses in two studies, safety (i.e., lack of increased TEAS rates) was comparable for rapid cycling vs. non-rapid cycling groups (Amsterdam et al. 2013; Altshuler et al. 2017).

## Long term treatment with antidepressants in bipolar disorder

Longer term studies examining the efficacy and safety of antidepressants, either as monotherapy with bipolar II patients or in combination with mood stabilizers in bipolar I and/or II patients are, understandably, few. Practice guidelines generally recommend only short term treatment with antidepressants in bipolar individuals (Yatham et al. 2013). This paucity of data is particularly unfortunate given the evidence that a substantial proportion of bipolar patients treated in the community have antidepressants as part of their maintenance regimen (Grande et al. 2013). Naturalistic studies suggested that a subgroup of patients that responded to a mood stabilizer plus an antidepressant did better if the antidepressant was included as part of a maintenance regimen, with lower depressive relapse rates and no increase in manic switches compared to the cohort whose antidepressants were withdrawn (Altshuler et al. 2001, 2003; Joffe et al. 2005).

In a long term (1–3 years) randomized study, continued antidepressant treatment with mood stabilizers was associated with somewhat less severe depressive symptoms and a mildly delayed time to depressive relapse with no difference in depressive episode prevalence (Ghaemi et al. 2010). Of note, the antidepressant continuation group showed no increase in switch rates compared to those whose antidepressants were withdrawn. However, increased cycling was observed in the subgroup of rapid cyclers treated with long term antidepressants (1.29 vs. 0.42 episodes per year, p = 0.04). Contrary to prediction, bipolar I patients showed a better long term response to maintenance antidepressants compared to bipolar IIs (Vohringer et al. 2015).

In a recent meta-analysis of the eleven studies examining the efficacy and safety of longer term antidepressants (> 4 months), antidepressants were superior to placebo in preventing depressive episodes (relative risk = 0.64, CI 0.49–0.83, p < 0.001), with or without mood stabilizers with no increase in manic/hypomanic episodes (Liu et al. 2017). Shorter studies (4–6 months) and longer term studies (6–24 months) showed similar findings.

Finally, a subtle and illustrative risk/benefit analysis was demonstrated in the Amsterdam and Shults study (2010). In this study, bipolar II patients who were short term responders to fluoxetine were randomly and blindly assigned to 1 year of treatment with either continued fluoxetine, lithium or placebo. Those subjects who continued on fluoxetine had fewer depressive relapses. There were no significant differences in a priori defined hypomanic episodes or mean mania rating scores across the three treatment groups. However, examining Young Mania Rating Scales (YMRS) ratings, it is clear that there was more mood fluctuation/variability in those treated with fluoxetine compared to the other two groups. Thus, the “cost” of remaining undepressed (with antidepressant monotherapy) was an increase in affective lability.

## Antidepressants in bipolar disorder: a clinical perspective

To summarize a relatively small but confusing literature:

- The efficacy of antidepressants in bipolar depression remains unproven.
- When added to mood stabilizers, antidepressants are not associated with increased switch (TEAS).
- No consistent evidence has demonstrated cycle acceleration in bipolar patients on modern antidepressants (especially with mood stabilizer co-treatment).
- Bipolar II patients may be treated safely (at least in the short term) with antidepressants.
- A subset of bipolar patients, both bipolar I and II, will need a maintenance regimen of mood stabilizers plus antidepressants and will not show mood instability with this regimen.

Given these conclusions, what principles should guide clinicians?

1. The key questions should not be simple dichotomous choices: are antidepressants effective for bipolar depression?, and are antidepressants harmful in bipolar patients? Rather, the right questions should be: For which bipolar patients will antidepressants be helpful? and for which bipolar patients will antidepressants be harmful? Additionally, the evidence of differential switch rates across antidepressant classes make the risk/benefit ratio depend on the specific antidepressant prescribed. Table 1 shows the risk factors for antidepressant-induced mania (TEAS). These risk factors do not define contraindications; e.g., although bipolar I patients are at higher risk to switch compared to bipolar II patients, some bipolar Is will respond to and be stable on antidepressants. These are risk factors, not universal truths. They should be used to create thoughtful algorithms of treatment. Thus, SSRIs and bupropion should, in general, be prescribed for bipolar patients prior to SNRIs due to higher switch rates associated with the latter—but some bipolar patients will need to be treated with an SNRI.

**Table 1:** Risk factors for treatment emergent affective switch

Bipolar I vs. bipolar II
Mixed depressive features
Rapid cycling
Antidepressant subtype-TCAs > venlafaxine (?other SNRIs) > SSRIs or bupropion
Substance abuse history, especially stimulants
2. Even with the predictors just noted, we cannot know about the risk/benefit ratio of antidepressants for any individual patient. Thus, an antidepressant may be prescribed with patient (and family, if involved) and psychiatrist aware of the potential risks. Both TEAS and cycle acceleration typically occur early in antidepressant treatment and can be monitored.
3. With bipolar II patients, most experts would agree that mood stabilizers should be prescribed first, before antidepressant monotherapy (although this generalization may change as we learn more). But some bipolar II patients will refuse a mood stabilizer as a first treatment for depression but will agree to take an antidepressant. In this situation, antidepressant monotherapy with proper education and monitoring is appropriate. With the exception of a few studies (e.g., Amsterdam and Shults 2010; Amsterdam et al. 2015), we have little data on the risks/benefits of longer term antidepressant monotherapy in bipolar II patients.
4. The subset of bipolar patients for whom a mood stabilizer/antidepressant combination is the optimal maintenance treatment regimen will be identified by:
  - Acute antidepressant efficacy.
  - One or more attempts to discontinue the antidepressant resulting in depressive relapse.

## Conclusion

The proper role of antidepressants in the treatment of bipolar depression continues to be a topic of deep clinical importance, marred by a lack of sufficient data with more heated opinions than consistent data-driven suggestions. We need both short (8–16 weeks) and longer term (6 month to 2 years) studies in both bipolar I and bipolar II patients (Gitlin 2012). Longer term studies are especially needed for bipolar II patients, given the efficacy and lack of harm seen in a few longer term (up to 1 year) antidepressant monotherapy studies (Amsterdam and Shults 2010; Amsterdam et al. 2015). Studies should include rapid cycling patients given the conflicting data on the potential harm associated with their use (Ghaemi et al. 2010; Amsterdam et al. 2013; Altshuler et al. 2017). Optimally, active comparators—lithium, lamotrigine, second generation antipsychotics should be included in the design of these studies. Until we have a larger data base, the question of antidepressants for bipolar depression will continue to generate more heat than light to the detriment of patients and clinicians alike.
